# Phenolic profiling and bioactivity assessment of *in vitro* propagated *Psidium cattleianum* Sabine: A promising study

**DOI:** 10.1016/j.heliyon.2024.e29379

**Published:** 2024-04-09

**Authors:** Eman M. El-Deeb, Heba E. Elsayed, Hanaa B. Ateya, Hussein S. Taha, Mohamed R. Elgindi, Doaa Abouelenein, Giovanni Caprioli, Kuei-Hung Lai, Ahmed M. Mustafa, Fatma A. Moharram

**Affiliations:** a Department of Pharmacognosy, Faculty of Pharmacy, October 6 University, Giza, Egypt; b Department of Pharmacognosy, Faculty of Pharmacy, Helwan University, Cairo, Egypt; c Department of Biochemistry and Molecular Biology, Faculty of Pharmacy, Helwan University, Cairo, Egypt; d Department of Plant Biotechnology, Biotechnology Research Institute, National Research Centre, Cairo, Egypt; e School of Pharmacy, University of Camerino, via Sant’ Agostino 1, Camerino, Italy; f Graduate Institute of Pharmacognosy, College of Pharmacy, Taipei Medical University, Taipei, Taiwan; g PhD Program in Clinical Drug Development of Herbal Medicine, College of Pharmacy, Taipei Medical University, Taipei, Taiwan; h Traditional Herbal Medicine Research Center, Taipei Medical University Hospital, Taipei, Taiwan; i Department of Pharmacognosy, Faculty of Pharmacy, Zagazig University, Zagazig, Egypt

**Keywords:** Anti-inflammatory, Anti-cancer, Antioxidant, HPLC-MS/MS, Micropropagation, Phenolics, Callus, Regeneration

## Abstract

*Psidium cattleianum* Sabine (strawberry guava) is an evergreen shrub that is grown as a fruiting hedge and has received significant consideration in the food and pharmaceutical disciplines. This study aims to set a promising protocol for *in vitro* propagation of *P. cattleianum*, along with profiling the phenolic content of the original plant (OP), induced callus (IC), and regenerated plantlets (RP) extracts, ultimately, evaluating their anti-inflammatory, antioxidant, and anticancer potential. Seeds were treated with commercial bleaching, HCl, and H_2_O_2_ to enhance the germination percentage and minimize the contamination percentage. Culturing sterilized leaf explants onto Murashige and Skoog (MS) medium supplemented with benzyl adenine (BA), 2,4-dichloro phenoxy acetic acid, and kinetin showed the best callus induction, while supplementation of MS media with BA, adenine sulfate, naphthalene acetic acid, and gibberellic acid activated regeneration. Augmentation of MS media with indol-3-butyric acid recorded the maximum rooting percentage. Finally, the obtained rooted shoots were successfully acclimatized in sand and peat moss soil. HPLC-MS/MS profiles of OP, RP, and IC showed a variety of phenolic metabolites. IC extract decreased the viability of MCF-7, HepG2, and K-562 cancer cell lines. Also, OP exhibits strong antioxidant activity. *P. cattleianum* and its RP are profound sources of phenolic compounds promoted for promising applications in the food and pharmaceutical industries.

## 1 Introduction

*Psidium cattleianum* Sabine, also acclaimed as strawberry guava, red guava, Chinese guava, cattley guava, or Araçá, is an exotic tropical shrub in the Myrtaceae family [1]. It is indigenous to Brazil and has been naturalized in different countries and many tropical islands due to the commercial potential of its fruits in the agriculture and food sectors [2]. *P. cattleianum* is known for its antimicrobial, antiproliferative [3], anti-inflammatory, antioxidant [4], anti-aging [5], and antidiabetic properties [6]. Its health benefits are linked to the high content of antioxidants such as vitamin C and phenolic phytoconstituents; consequently, it is a valuable quarry for the pharmaceutical industry [3]. During the past few decades, transpiring biological techniques for the micropropagation and tissue culture of plant cultivars have been adopted by several researchers [7]. Micropropagation refers to an optimized *in vitro* approach for plant propagation in terms of time management and large-scale production. Micropropagation is commonly composed of three stages: establishment, multiplication, and rooting [8,9] later, Hartmann and co-workers [10] added acclimatization as a fourth stage. In the coming years, this technology is expected to contribute more, both on its own and in conjunction with the use of molecular biology. For instance, micropropagation has made significant contributions to the production and improvement of several crops, such as yellow guava (*Psidium guajava* L.) [7].

Tracking, identifying, and quantifying phytoconstituents in a complex plant extract is a challenging task. Nevertheless, today, with the availability of various state-of-the-art identification techniques, specific stationary phases, and detectors, this mission is affordable and can solve any identification problem. Among the most important and standard techniques is high-performance liquid chromatography, hyphenated to tandem mass spectrometry (HPLC-MS/MS) [11]. The HPLC fingerprint using natural authentic standards is a comprehensive method approved by the FDA for the assessment of the quality and reliability of plant extracts [12]. In this context, the implementation of HPLC/MS has been previously reported to investigate the phenolic phytoconstituents from *P. cattleianum* leaves collected from Brazil, Korea, and Mexico [[13], [14], [15], [16], [17], [18]]. In addition to the prior analysis of *P*. *cattleianum* fruit at three different ripening stages by Schulz et al. [19] who identified twenty-three phenolics, among which catechin, isoquercitrin, quercetin, gallic acid, and syringic acid were detected in significant concentrations in all the evaluated stages. As for the unprecedented agronomical, horticultural, and health benefits of *P. cattleianum,* designing a time-saving protocol to extend the production of the elite *P. cattleianum* genotype with the conservation of its bioactive phenolic constituents is a dire target.

Herein, the current research aims to set a promising protocol for *in vitro*-propagation of *P. cattleianum* Sabine. Moreover, profiling the phenolic compounds from different chemical classes, such as phenolic acids, flavan-3-ols, flavonols, dihydrochalcone, and flavanone using HPLC-MS/MS in calli cultures and regenerated plantlets in comparison to the mother plant. Ultimately, the antioxidant, anti-inflammatory, and cytotoxic capacities of the three extracts have been compared.

## 2 Results and discussion

### 2.1 *In vitro* propagation of *Psidium cattleianum* Sabine

Worldwide, propagation methods have been implemented for several guava cultivars to increase their production in tropical and subtropical regions [7]. In the current study, we set a prime micropropagation protocol for *P. cattleianum* Sabine, a commercially important, tropical tree that can provide a panel of nutrients and phytochemicals with substantial biological functions [3]. Furthermore, the phenolic metabolites in the regenerated plantlet and the induced callus were identified and quantified in comparison to the original, mother *cattleianum*.

#### 2.1.1 Sterilization and seed germination

Our results in Table 1 show the effect of the sterilization process on seed germination and development contamination percentages. Treatment of the seeds with 15 % Clorox® commercial bleach for 15 min, followed by soaking in 15 % HCl for 24 h, then in 10 % H_2_O_2_ for 48 h (treatment T4) resulted in the highest percentage of seed germination (63.43 ± 6.18 %) and the lowest percentage of contamination (34.67 ± 7.11 %). The obtained results followed a prior study by Freire and co-workers [20] who claimed the effect of H_2_SO_4_ scarification on the germination of *P. cattleianum* seeds. These results may be due to the ability of acids in the penetration of *P. cattleianum* seeds which have an impermeable integument with a hard consistency [21] leading to the seed's low imbibition rate and consequent physical dormancy [22]. Meanwhile, 15 % H_2_O_2_ has a crucial role in removing air voids for efficient surface sterilization [23]. As well as data in Table 2 clearly shows that half salt strength of MS medium (M2) was supreme in enhancing seed germination, percentage seed development, and shoot length of seedlings by 75.66 ± 4.97 %, 34.33 ± 4.63 %, and 3.56 cm, respectively. On the other side, WPM-free, basal (M5) was the least favorable medium as it possessed 24.33 ± 3.75 % and 10 ± 3.28 % of seed germination and development, respectively. The obtained results were in line with Usman et al. [24] who previously reported that half basal MS was the best medium for the germination of yellow guava (*Psidium guava*), one of the most ubiquitous *Psidium* species worldwide.

**Table 1:** Effect of different treatments (T1-T4) on *in vitro* seed germination and contamination percentages of *P. cattleianum*.

Treatments	Composition	%Germination	% Contamination
**T1**	10 % Clorox® commercial bleaching/15 min	4.76 ± 0.70^c^	92.67 ± 0.77^a^
**T2**	15 % Clorox® commercial bleaching/15 min	7.86 ± 1.21^c^	83.13 ± 2.87^a^
**T3**	15 % Clorox® commercial bleaching, soaking in 10 % HCl/24 h, then 10 % H_2_O_2_/48 h	44.13 ± 4.16^b^	55.20 ± 3.76^b^
**T4**	15 % Clorox® commercial bleaching, soaking in 15 % HCl/24 h, then 10 % H_2_O_2_/48 h	63.43 ± 6.18^a^	34.67 ± 7.11^c^

Data are represented as means ± SE. Values followed by different superscripts in the same column are significantly different according to Duncan's Multiple Range Test (DMRT) at *p* ≤ 0.05. Data collected after 28 days.

**Table 2:** Effect of different types of media (M1–M6) on *in vitro* seed germination, development percentage of *P. cattleianum,* and shoot length of the seedlings after four weeks of germination.

Media	Composition	%Germination	%Development	Shoot length (cm)
**M1**	MS basal (free from growth regulator)	60.33 ± 2.72^b^	21.33 ± 1.76^b^	3.13 ± 1.45 ^b^
**M2**	Half strength, MS	75.66 ± 4.97^a^	34.33 ± 4.63^a^	3.56 ± 0.08^a^
**M3**	LS basal	39.33 ± 2.72^c^	17.66 ± 2.33^b,c^	2.30 ± 0.11^c^
**M4**	Half strength, LS	46 ± 3.60^c^	19.66 ± 3.28^b^	2.80 ± 0.05 ^b^
**M5**	WP basal	24.33 ± 3.75^d^	10 ± 3.28^c^	1.53 ± 0.08^d^
**M6**	Half strength, WP	27.33 ± 3.17^d^	13.3 ± 0.88^b,c^	1.96 ± 0.20^c^

MS: Murashige and Skoog, LS: Linsmaier and Skoog, WP: Woody plant. Data are represented as means ± SE. Values followed by different superscripts in the same column differ significantly according to Duncan's Multiple Range Test (DMRT) at p ≤ 0.05. Data collected after 28 days.

#### 2.1.2 Callus induction and growth kinetics

**Figure (1):**
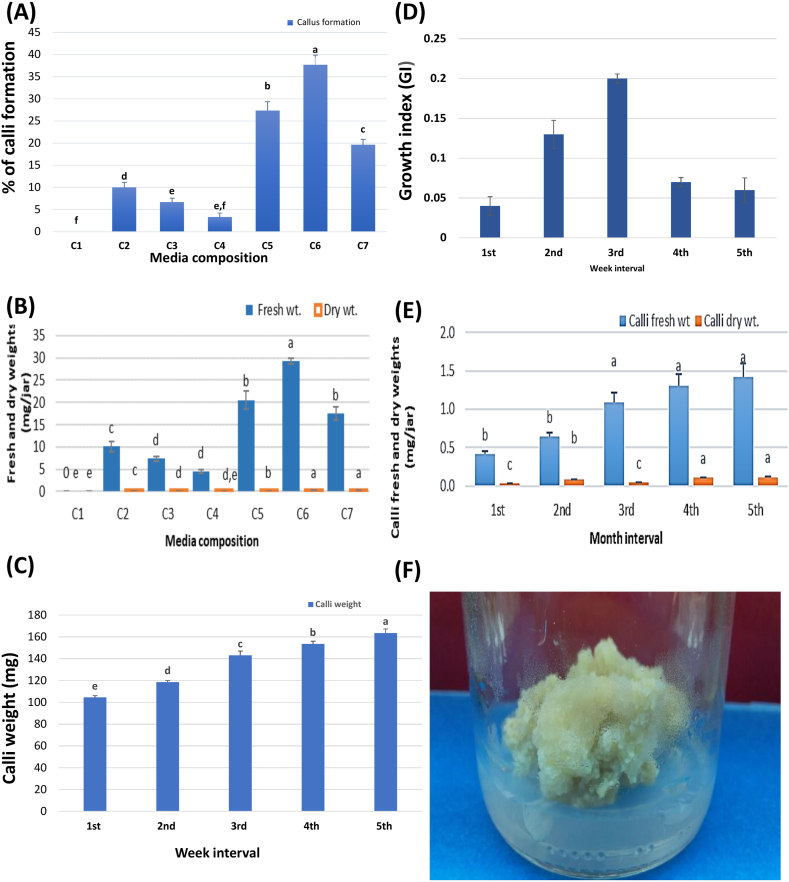
Effect of MS-media composition on calli formation percentage (A), calli fresh and dry weights (B). Progress of calli weights across five weeks (C). Growth kinetic index during five weeks (D). Calli fresh and dry weights (g/jar) during five months (E). Calli production after five sub-cultures on (C6) medium, incubated under light condition 16/8 h (F). **Where**: C1: MS free, basal, C2: MS+ 1 mg/L BA, C3: MS+ 1 mg/L 2,4-D, C4: MS+ 1 mg/L Kin, C5: MS+1 mg/L BA +1 mg/L 2,4-D + 0.2 mg/L Kin, C6: MS+3 mg/L BA +1 mg/L 2,4-D + 0.2 mg/L Kin, C7: MS+5 mg/L BA +1 mg/L 2,4 D + 0.2 mg/L Kin. BA: Benzyl adenine; 2,4-D: 2,4-Dichlorophenoxy acetic acid; Kin: kinetin. Data are represented as means ± SE. Values followed by different superscripts in the same column differ significantly according to Duncan's Multiple Range Test (DMRT) at p ≤ 0.05.

MS medium fortified with 3 mg/L of BA in combination with 1 mg/L 2,4-D and 0.2 mg/L Kin (C6-Table 3) achieved the highest percentage of callus formation from leaf explants (37.66 ± 2.18 %), with subsequent increased callus fresh, and dry weights to 29.33 ± 0.66 and 0.22 ± 0.01 mg/culture, respectively (Fig. 1A and B). On the contrary, supplementation of MS medium with Kin (1 mg/L Kin) only (C4, Table 3) reduced the percentage of callus formation (3.33 ± 0.88 %) and fresh and dry weights (4.46 ± 0.38 and 0.02 ± 0.01 mg/culture, respectively, Table 3). As a general observation, all culture media, under light conditions, induced calli formation with a variable percentage, while no results were observed with incubation under dark conditions. Four weeks later, most of the leaf explants have produced a friable yellowish-green callus with a notable weight increase proportionally across weeks. Calli's fresh weight after the first week was recorded 104.6 ± 1.49 mg/culture and reached 163.6 ± 3.69 mg/culture by the fifth week (Fig. 1C) with the maximum growth index (GI 0.2, Fig. 1D) being observed in the third week. Callus production has been based on a combination of auxin and cytokinin, as both mutually control the process of cell division. Auxins play a vital role in the stimulation of proteins in the cell cycle as related cyclin-dependent kinases (cdc2/cdk2). Furthermore, the applications of auxins with cytokinins synergistically increase the activity of cdc2/cdk2 kinases [25], hence producing more callus mass whereas the optimum ratio is essential for maximum results [26]. BA as a potent cytokinin can promote culture growth and development by stimulating cell division [27], while 2,4-D, and kinetin (the most popular auxins) have a role in promoting callogenesis [28]. A combination between these different growth regulators may outperform their effect which agrees with our results and promotes medium C6 as the best medium for callus induction. Calli production was monitored over a long culture period (5 months) using medium C6 (the medium that showed the best short-term calli production). The results showed that calli fresh and dry weights were increased proportionally by increasing the incubation period and reached their maximum values by the 5^th^ month (Fig. 1E and F).

**Table 3:** Effect of various growth regulators supplemented to MS media, individually or in combination, on calli formation %, calli fresh and dry weights (mg/culture) of *P. cattleianum*.

Media	Composition	% Calli	Fresh weight	Dry weight
**C1**	MS free, basal	0^f^	0^e^	0^e^
**C2**	MS+ 1 mg/L BA	10 ± 1.15^d^	10.10 ± 1.15^c^	0.07 ± 0.01^c^
**C3**	MS+ 1 mg/L 2,4-D	6.66 ± 0.88^e^	7.33 ± 0.54^d^	0.06 ± 0.01^c,d^
**C4**	MS+ 1 mg/L Kin	3.33 ± 0.88^e,f^	4.46 ± 0.38^d^	0.02 ± 0.01^d,e^
**C5**	MS+1 mg/L BA +1 mg/L 2,4-D + 0.2 mg/L Kin	27.33 ± 2.02^b^	20.53 ± 1.97^b^	0.13 ± 0.02^b^
**C6**	MS+3 mg/L BA +1 mg/L 2,4-D + 0.2 mg/L Kin	37.66 ± 2.18^a^	29.33 ± 0.66^a^	0.22 ± 0.01^a^
**C7**	MS+5 mg/L BA +1 mg/L 2,4 D + 0.2 mg/L Kin	19.66 ± 1.20^c^	17.57 ± 1.50^b^	0.19 ± 0.01^a^

BA: Benzyl adenine; 2,4-D: 2,4-Dichlorophenoxy acetic acid; Kin: kinetin. Data are represented as means ± SE. Values followed by different superscripts in the same column differ significantly according to Duncan's Multiple Range Test (DMRT) at p ≤ 0.05. Incubation in the dark for 3 days then transferred to light conditions at 26 ± 1 °C for 21 days. Each treatment consisted of three replicates (jars). Each replicate contained three explants (leaves).

#### 2.1.3 Indirect shoot regeneration

**Figure (2):**
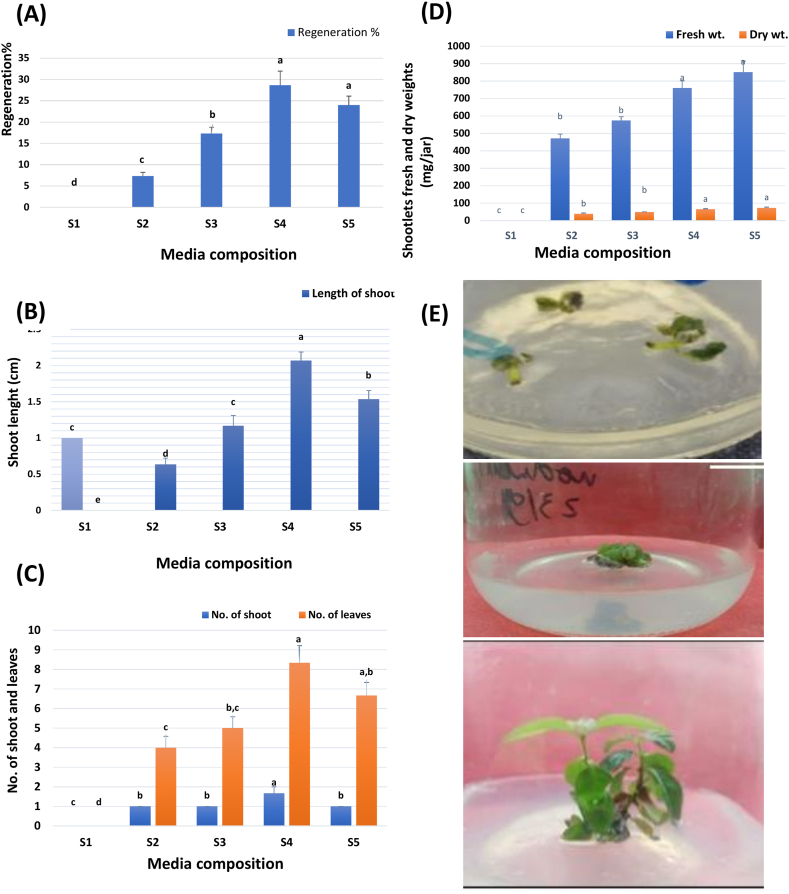
Effect of MS-media composition on regeneration percentage (A), shoot length (B), no. of shoot and leaves/shoot (C), fresh and dry weights of regenerated plantlets (D). E(I: IV) Indirect plant regeneration; beginning from callus formation from leaf explants on C6 medium (I), greening of callus before regeneration of shoots on S4 medium (II), shoot differentiation from callus on S4 (III) and regenerated plantlet from *P. cattleianum* leaf calli cultured on S4 medium*, incubated* for 4 weeks under light conditions (16/8 h). **Where: C6:** MS+3 mg/L BA +1 mg/L 2,4-D + 0.2 mg/L Kin, **S1**: MS free, basal, **S2**: MS+ 1 mg/L BA+ 160 mg/L AS + 0.5 mg/L NAA+ 0.2 mg/L GA_3,_**S3**: MS+ 2 mg/L BA+160 mg/L AS+0.5 mg/L NAA+0.2 mg/L GA_3,_**S4**: MS+ 3 mg/L BA+160 mg/L AS+0.5 mg/L NAA+0.2 mg/L GA_3_, **S5**: MS+ 3 mg/L BA+160 mg/L AS+0.5 mg/L NAA+0.2 mg/L GA_3_. BA: Benzyl adenine; AS: Adenine sulfate; NAA: Naphthalene acetic acid, GA3: Gibberellic acid; Data are represented as means ± SE. Values followed by different superscripts in the same column differ significantly according to Duncan's Multiple Range Test (DMRT)at p ≤ 0.05.

Regarding the regeneration experiment, data showed a significant difference among the tested media (S1–S5, Fig. 2A–E, and Table S1), except for the number of regenerated shoots which showed no significant difference (Fig. 2C). Overall, the data, revealed that supplementation of MS medium with 3 mg/L BA in combination with 160 mg/L adenine sulfate, 0.5 mg/L NAA, and 0.2 mg/L GA3 (medium S4) recorded the highest values for most of the investigated parameters; regeneration percentage (28.6 ± 3.28 %), length of shoots (2.06 ± 0.12 cm), the average number of shoots (1.66 ± 0.33), and the relative number of leaves/shoot (8.33 ± 0.88). Although medium S5 fortified with a high concentration of BA up to 5 mg, it recorded a decreased percentage of regeneration (24 ± 2.08 %) and increased fresh and dry weights (851.73 ± 62.90 and 73.13 ± 4.38 mg/culture) respectively, compared to medium S4 (Table S1, Fig. 2D). This result follows those reported by Freire et al. [29] who mentioned that in Myrtaceae explants a higher concentration of phytohormones could be considered as phytotoxic. In this regard, Rai and his research team [30] showed that using BA in a concentration of 1 mg/L achieved a high number of buds of *P. guajava* while increasing the concentration to 2 mg/L induced a sharp decrease in bud formation. Other research groups [31,32] have reported the same observation they found that high concentrations of cytokinin have deleterious effects on the multiplication of shoots in *Aspilia africana* and *Ruta chalepensis*, respectively. BA as an adenine cytokinin promotes cell division in plant tissues; hence increasing its level will increase the number of cells, subsequently with cell contents, and the percentage of absorbed water in the cell tissues. In all, this led to increasing the fresh and the dry weights [33,34].

#### 2.1.4 Roots induction

**Figure (3):**
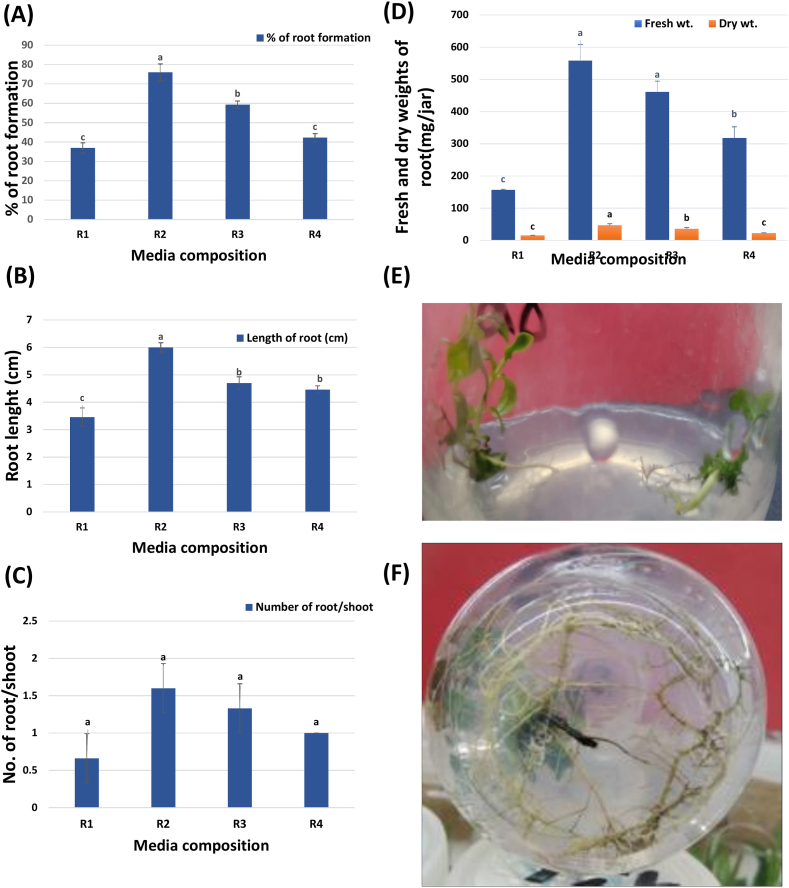
Effect of MS-media composition on the percentage of root formation (A), root length (B), no. of root/shoot (C), fresh and dry weights of roots (D), regenerated shootlets (E), developed roots (F). Developed roots from regenerated shootlets cultured on (R2) medium after two weeks (I) and four weeks (II)*,* incubated for 4 weeks under light conditions (16/8 h). **Where: R1**: MS free, basal, **R2**: MS+1 mg/L IBA, **R3**: MS+ 1mg/I AA, **R4**: MS + MS+1mg/L IBA: Indole-3-butyric acid, IAA: Indole-acetic acid, NAA: Naphthalene acetic acid. Data are represented as means ± SE. Values followed by different superscripts in the same column differ significantly according to Duncan's Multiple Range Test (DMRT) at p ≤ 0.05.

Data in Figures (3A-F) demonstrated that MS media supplemented with 1 mg/L IBA (Medium R2) resulted in the highest percentage of root formation (76 ± 4.35 %), root length (6 ± 0.17 cm), and relative number of roots/shoot (1.66 ± 0.33). Moreover, it recorded the highest values for the calculated fresh and dry weights 557.83 ± 50.3 and 46.80 ± 5.11 mg/culture, respectively (Table S2). On the other hand, MS-free basal media (R1), achieved values that were nearly half as low as R2 (Table S2). These results were in line with those previously reported by Mohamed-Yasseen and co-workers [35] on the root generation of *P. guava*. Their study showed that the highest percentage of root generation was achieved by including IBA in the adopted media.

#### 2.1.5 Acclimatization stage

**Figure (4):**
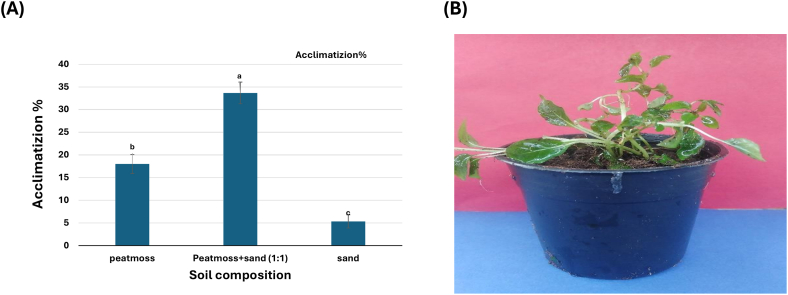
(A) Percent acclimatization of *P. cattleianum* plantlets to different pot soils. (B, C) Acclimatization of micropropagated *P. cattleianum* plantlets transplanting on Peat moss: sand (1:1) after one month and three months.

Fig. 4A and B and Supplementary Table S3 clearly show a highly significant difference between different types of applied potting media. For instance, the combination of peat moss and sandy soils in an equivalent ratio recorded 33.6 ± 2.4 % of acclimatized plantlets (33 acclimatized plantlets). On the other side, adopting sandy soil alone recorded the lowest percentage (5.3 ± 1.4 %). Our results agreed with those previously reported [36] as the combination between peat moss and sand (1:1) significantly increased the survival rate and acclimatization of *Gynerium sagittatum* (arrow cane) when transferred to *ex vitro* conditions.

### 2.2 Extraction, quantification, and identification of the phenolic metabolites

The dried *P. cattleianum* original plant (OP), induced callus (IC), as well as the regenerated plantlet (RP) (≈500 mg each), were separately extracted using 80 % aqueous methanol to yield 235, 140, and 180 mg dry extracts, respectively. The three extracts were subjected to comprehensive phytochemical analysis, which included the quantification of the total phenolic, tannin, and flavonoid contents in addition to the identification of their phenolic metabolites using HPLC-MS/MS. The quantification data (Table 4) showed that the RP possessed high phenolics, tannins, and flavonoids contents, calculated as 64.69 ± 0.59 mg GAE/g, 71.03 ± 0.65 mg TAE/g, and 1.46 ± 0.08 mg CE/g dry wt., respectively. The values were largerly equivalent to the OP (Table 4), while being three-fold higher than the callus (26.17 ± 1.44 mg GAE/g, 28.83 ± 4.08 mg TAE/g, and 0.41 ± 0.01 mg CE/g dry wt.). This is a promising indication, as the regenerated plantlets, at least in part, maintained the phenolic content of the OP. Meanwhile, the callus stage expressed low but significant values because it is merely an unorganized growing and dividing mass that will retain the full capacity of the biosynthetic machinery after being differentiated and transformed into a regenerated plantlet. Subsequently, each extract was subjected to tandem HPLC-MS profiling following the acquisition parameters stated in Table S4. Thirty-eight standard phenolics were adopted, of which twenty-eight compounds were identified in the OP, twenty-five in each of the RP and IC representing 128.261, 58.735, and 30.886 mg/g, phenolic content respectively (Table 5). The values exhibited great similarity with the quantified total phenolics, for the IC and RP, using the Folin-Ciocalteu method, while the OP displayed larger values. The reason could be related to the advantage of the quantification by HPLC-MS/MS, which is more specific and accurate, due to using polyphenol references. Thus, the total polyphenol content was calculated precisely as the sum of all detected polyphenol standards, not on one single reference, and presented as gallic acid equivalents [37]. After a keen examination of the HPLC results, we deduced that most of the identified phenolic metabolites fall under the categories of phenolic acids (98.453–27.735 mg/g), flavonols (27.994–2.489 mg/g extract) as well as, flavan-3-ols (1.603–0.0047 mg/g). Meanwhile, dihydrochalcones, anthocyanins, and flavanone were signified in the tested samples at low microgram levels. For instance, hesperidin is the only noticed flavanone (375.00–12.84 μg/g), phloridizin is the only dihydrochalcones detected in all samples, while phloretin traced only in the OP. Meanwhile, delphinidin-3-*O*-galactoside is the only identified anthocyanin (9.36–2.62 μg/g). Moreover, the documentation of ellagic acid, gallic acid, isoquercitrin, quercetin, hyperoside, catechin, vanillic acid, myricetin, coumaric acid, caffeic acid, chlorogenic, ferulic, and syringic acid in the OP was expected and matched with the prior studies published about the *P. cattleianum* collected from Brazil, Korea, and Mexico [[13], [14], [15], [16], [17], [18]]. Although there is a variation in the quantified percentages, and in the presence of unprecedented metabolites as neo-chlorogenic, delphinidine-3-*O*-galactoside, rutin, kaempferol-3-*O*-glucoside, and isorhamnetin in *P. cattleianum* (OP) from Egypt. Yet the cause of these differences may be due to the distinctive geographical region, the handling protocol after collection, the conditions of extraction, the methods of detection, and identification [18]. We also observed the distinction of IC by the biosynthesis of large amounts of vanillic acid (5216.741 μg/g), ferulic acid (11.487 μg/g), hesperidin (375.002 μg/g), and *trans*-cinnamic acid (275.238 μg/g) in comparison to the RP. The reason may be linked with the consumption of these metabolites by the RP for the biosynthesis and formation of various, sophisticated phenolic metabolites. For instance, cinnamic acid (abundant in the IC) is consumed by the RP for the biosynthesis of coumaric acid (noticeable in the RP), meanwhile, the latter is a precursor to the biosynthesis of a panel of flavonoids in both RP and OP [38]. Moreover, we cannot neglect the impact of the various growth regulators (supplemented in the culture media) on the biosynthetic pathway and transportation of some phenolic metabolites [39]. On the other side, the RP was marked by high levels of *p*-hydroxybenzoic acid (284.760 μg/g), 3,5 caffeoylquinic acid (201.267 μg/g), and kaempferol (343.588 μg/g). Kaempferol, for instance, is a key precursor for the biosynthesis of quercetin and other quercetin-based glycosides found in the *P*. *cattleianum* OP [40]. Moreover, the combination of cytokinins and auxins such as BA and IAA has a synergistic effect on most of the flavonoid's content and significantly increases the production and accumulation of some phenolic acids [41].

**Table 4:** Equivalent total phenolic (TP), tannin (TT), and flavonoid (TF) contents of *P. cattleianum* in mother plant, callus, and regenerated plantlets.

Explant	TP	TT	TF
**Original plant**	77.71 ± 1.59	85.32 ± 1.75	1.71 ± 0.06
**Induced Callus**	26.17 ± 1.44	28.83 ± 4.08	0.41 ± 0.01
**Regenerated plantlets**	64.69 ± 0.59	71.03 ± 0.65	1.46 ± 0.08

Concentrations are expressed as mg standard equivalent/g extract.

**Table 5:** Phenolic compounds (μg/g) identified in *P. cattleianum* 80% aqueous methanol extracts of original plant (OP), induced Callus (IC), and regenerated plantlet (RP) by HPLC-MS/MS (Relative SD ≤ ±5.3).

No.	Compound	OP	IC	RP
**Phenolic acids**				
**1**	Gallic acid	39367.382	10817.248	18521.090
**2**	Neochlorogenic acid	42.795	n.d.	n.d.
**3**	Chlorogenic acid	156.081	12.905	64.868
**4**	*p*-Hydroxybenzoic acid	259.833	262.326	284.760
**5**	3-Hydroxy benzoic acid	23.538	20.254	30.289
**6**	Caffeic acid	68.406	28.362	20.657
**7**	Vanillic acid	406.222	5216.741	662.783
**8**	Syringic acid	59.020	49.886	21.079
**9**	*p*-Coumaric acid	261.702	99.984	263.463
**10**	Ferulic acid	3.093	11.487	9.499
**11**	3,5-Dicaffeoylquinic acid	174.094	20.838	201.267
**12**	Ellagic acid	57631.430	11195.391	28905.454
**Flavonoids**				
A) **Anthocyanins**				
**13**	Delphinidin-3,5-*O*-diglucoside	n.d.	n.d.	n.d.
**14**	Delphinidin-3-*O*-galactoside	9.365	2.626	5.928
**15**	Cyanidin-3-*O-*glucoside	n.d.	n.d.	n.d.
**16**	Petunidin-3-*O-*glucoside	n.d.	n.d.	n.d.
**17**	Pelargonidin-3-*O-*rutinoside	n.d.	n.d.	n.d.
**18**	Pelargonidin-3-*O*-glucoside	n.d.	n.d.	n.d.
**19**	Malvidin-3-*O-*galactoside	n.d.	n.d.	n.d.
B) **Flavonols**				
**20**	Rutin	43.249	28.293	16.514
**21**	Isoquercitrin	5922.728	461.440	1149.338
**22**	Quercitrin	9267.113	1101.772	1804.200
**23**	Myricetin	311.869	16.631	102.345
**24**	Kaempferol-3-*O*-glucoside	186.378	51.729	118.150
**25**	Quercetin	4498.159	248.113	4401.123
**26**	Isorhamnetin	40.220	14.789	121.976
**27**	Hyperoside	7563.529	547.305	1359.414
**28**	Kaempferol	160.754	19.791	343.588
C) **Flavan-3-ols**				
**29**	Catechin	1589.621	4.782	159.228
**30**	Epicatechin	13.409	0.000	0.000
**31**	Procyanidin B2	n.d.	n.d.	n.d.
**32**	Procyanidin A2	n.d.	n.d.	n.d.
D) **Dihydrochalcones**				
**33**	Phloridzin	104.940	3.064	6.850
**34**	Phloretin	1.166	0.000	0.000
E) **Flavanones**				
**35**	Hesperidin	12.837	375.002	24.687
**36**	Naringin	n.d.	n.d.	n.d.
**Stilbenes**				
**37**	Resveratrol	n.d.	n.d.	n.d.
**Non-Phenolic acids**				
**38**	*Trans*-cinnamic acid	82.677	275.238	136.790
	Total Anthocyanins	9.36	2.63	5.93
	Total Flavonols	27994.00	2489.86	9416.65
	Total Flavan-3-ols	1603.03	4.78	159.23
	Total Dihydro chalcones	106.11	3.06	6.85
	Total Flavanone	12.84	375.00	24.69
	Total Phenolic acids	98453.60	27735.42	48985.21
	**Total phenolic content**	128261.61	30886.00	58735.34

### 2.3 The biological assessment of *P. cattleianum* extracts

The 80% aqueous methanol extracts derived from *P. cattleianum* OP, IC, and RP, were separately assessed for their potential effect on the viability of MCF-7, HepG-2, and K-562 cancer cells. The cells were treated with a series of concentrations (0–1000 μg/mL) from the tested extracts for 24 h and the percent viability was determined using the MTT assay. The results, displayed in Table 6 and Fig. S1, indicated that IC extract displayed the best cytotoxic effect (IC_50_ 242.29–438.44 μg/mL), in comparison to other tested extracts, with the most significant effect observed on HepG-2. Scanning the available literature, we deduced that the methanol extract of *P*. *cattleianum* fruit is non-toxic to MCF-7 (breast cancer cells) and Caco-2 (colon cancer cells), however, it may affect the cancerous cells' proliferation [3]. They correlated the weak cytotoxicity to the high phenolic content of the fruit extract because polyphenols are potent radical scavengers and thus prevent the cytotoxicity cascade through potential antioxidant mechanisms [3]. Concurrently, this may explain our results in which the IC possessed the least phenolic content which is linked to the highest cytotoxicity. Likewise, the anti-inflammatory potential of the 80 % aq. methanol extract of OP, IC, and RP was assessed using the lipopolysaccharide (LPS)-induced macrophage inflammatory model. The extracts were primarily screened for their effect on the viability of RAW 264.7 (macrophage-like cells) using the MTT assay, and then the sub-cytotoxic effective concentrations were determined. The results (Table 7) revealed that all tested extracts displayed weak toxicity with IC_50_ equivalent to 443.25 ± 13.2 μg/mL (OP), 546.37 ± 22.7 μg/mL (IC), and 783.25 ± 17.4 μg/mL (RP). subsequently, LPS-stimulated macrophages were treated separately with the tested extracts (OP, IC, and RP) at ½ IC_50_ concentrations, to be sure that the effect was due to a pro- or anti-inflammatory impact rather than a cytotoxic effect. At the end of the treatment period, the levels of secreted cytokines such as IL-10, IL-6, and TNF-*α* in the supernatant were determined. Cytokines are regulators of host responses to infection, immune responses, inflammation, and trauma. Some cytokines act to make the disease worse (proinflammatory), whereas others serve to reduce inflammation and promote healing (anti-inflammatory) [42]. Data in Table 7 shows that the secretion of the pro-inflammatory cytokine, IL-6, was significantly reduced by 16 % and 13.5 % upon incubation with OP and IC, respectively when compared to the positive control LPS-induced group (IC_50_ 596.2 ± 7.2 pg/mL). However, the RP showed a non-significant effect on reducing the IL-6 leakage. Furthermore, when compared to the positive control, OP and IC reduced TNF-*α* (a pro-inflammatory cytokine) concentrations by 22 % and 10 %, respectively (Table 7), whereas RP did not significantly reduce TNF-*α* levels after 24 h of treatment. On the other side, interleukin-10 (IL-10) is a key anti-inflammatory cytokine that can inhibit pro-inflammatory responses of both innate and adaptive immune cells [43]. Hence, stimulating its release will concurrently decrease the inflammatory cascade. In the current investigation, IL-10 secretion was enhanced by 48 %, 32 %, and 17.5 % after treatments with OP, IC, and RP, respectively, compared to the positive control (Table 7). Overall, OP and IC extracts inhibited IL-6 and TNF-*α* secretion and stimulated IL-10 release in RAW 264.7 cells, resulting in mild to moderate anti-inflammatory activity. We previously discussed that the OP extract is exclusively rich in ellagic acid, gallic acid, and epicatechin. These phenolic metabolites are reported to be potent anti-inflammatory, suppressing IL-6 and TNF-*α* and increasing IL-10 production *via* NF-_k_B nuclear translocation in the whole blood-stimulated system [44,45]. Whereas we report that the IC extract was pioneered with vanillic acid and hesperidin. Interestingly, prior studies showed that hesperidin reduced pro-inflammatory cytokine levels at least partially *via* the miRNA-132 pathway [46], while vanillic acid inhibits LPS-induced TNF-*α* and IL-6 production by suppressing NF-_K_B in lipopolysaccharide-stimulated mouse peritoneal macrophages [47]. Lastly, we pinpointed the extract of the RP by its substantial content of phenolics, especially kaempferol which is noticed to reduce the intensity of inflammatory processes by increasing the secretion of anti-inflammatory cytokine IL-10 [48].

**Table 6:** IC_50_ of *P. cattleianum* extracts, original plant (OP), induced callus (IC), regenerated plantlets (RP), in the viability assay on HepG-2 (hepatic), MCF-7 (breast), and K-562(leukemia) cancer cell lines.

	IC_50_ ± SD (μg/mL)		
**Tested samples**	HepG-2	MCF-7	K-562
**OP**	659.03 ± 16.23	862.20 ± 19.48	944.88 ± 27.95
**IC**	242.29 ± 3.47	377.004 ± 12.34	438.44 ± 10.21
**RP**	>1000	>1000	>1000

**Table 7:** IC_50_ of *P. cattleianum* OP, IC, and RP extracts, in the MTT viability assay (mean ± SD μg/mL), inflammatory markers (mean ± SD pg/mL), and DPPH assay in the lipopolysaccharide-induced RAW 264.7 macrophage cells.

Tested samples	IC_50_ ± SD	½ IC_50_	IL-6 ± SD	TNF-*α* ± SD	IL-10 ± SD	DPPH
**OP**	443.25 ± 13.2	221.6	501 ± 2.5 *** (16 %)	318.8 ± 10.9 ** (22 %)	69.4 ± 3.02 ** (48 %)	8.9 ± 0.7
**IC**	546.37 ± 22.7	273.2	515 ± 6.6 *** (13.5 %)	372.2 ± 5.7 ** (10 %)	62.02 ± 3.6 * (32 %)	95.1 ± 3.3
**RP**	783.25 ± 17.4	391.6	587 ± 11.1 (1.5 %)	396.6 ± 7.4 (3 %)	55.4 ± 3.1 * (17.5 %)	23.7 ± 1.3
**Negative control**			161.8 ± 12.1	19.3 ± 7.5	28 ± 3.4	
**Positive control**			596.2 ± 7.2	408.9 ± 2.8	47.11 ± 1.4	
**Trolox**						6.2 ± 0.6

Values between parenthesis represent the percent of inhibition.

Due to the antioxidant properties of phenolic metabolites, they can scavenge free radicals such as reactive oxygen species [49]. Herein, the antioxidant effect of *P. cattleianum* extracts was estimated in terms of the extracts' ability to scavenge DPPH. DPPH radical scavenging assay is among the most frequently used methods and offers the first approach for evaluating phenolics’ antioxidant ability [50]. DPPH is a stable chromogen radical with a deep purple color, and the assay is based on the electron donation of antioxidants to neutralize the DPPH radical. The reaction is accompanied by a color change of the DPPH and the discoloration acts as a marker of antioxidant efficiency. Subsequently, the lower the IC_50_ is the higher the antioxidant potential [51]. The results of the DPPH scavenging revealed that the OP exhibited potent antioxidant power with IC_50_ 8.9 ± 0.7 followed by RP (23.7 ± 1.3) in comparison to Trolox (6.2 ± 0.6), while IC showed the least activity. According to the literature, there are diverse theories that correlate the strength of the antioxidant effect of phenolic metabolites with the applied *in vitro* assay. The DPPH protocol, seems to be influenced by the first and third Bors standards, which linked the radical scavenging property to specific structural features [52,53]. The first Bors criteria linked the presence of a catechol group on the B-ring with high stability of the antioxidant radical, while the third correlated the presence of OH groups at positions 3 and 5 in combination with the 4-oxo group enables electron delocalization *via* hydrogen bonds [52,53]. The aforementioned features are almost found in most of the identified flavonoids in the three tested samples, so the variations in the radical scavenging results between the three extracts, may be linked with the structural features of their phenolic constituents.

## 3 Materials and methods

### 3.1 Design of the micropropagation protocol

#### 3.1.1 Plant material

*Psidium cattleianum* Sabine mature fruits were obtained from Mazhar Botanical Garden, Cairo, Egypt, in August 2019. Botanical identification was achieved by Dr. Trease Labib, and a voucher specimen was allotted to the Pharmacognosy Department, Faculty of Pharmacy, Helwan University, Egypt, and assigned as 02Pca/2019. The seeds, as explants, were detached from the mature fruits and used for the establishment of a micropropagation protocol in the Plant Biotechnology Department, Genetic Engineering and Biotechnology Research Institute, National Research Center, Cairo, Egypt.

#### 3.1.2 Sterilization and seed germination

*P. cattleianum* seeds were thoroughly washed under running tap water, then with liquid detergent for 15 min, followed by immersion in 70 % ethanol for 10 s, and finally rinsed three times with sterile, distilled water. Subsequently, the seeds were surface sterilized under air laminar flow, then rinsed four times with sterile, distilled water. Next, the seeds were treated with either 10–15 % Clorox® commercial bleaching alone or in combination with 10–15 % HCl and 10 % H_2_O_2_ (T1-T4, Table 1) to enhance the germination process and minimize the possibility of contamination. Sterilized seeds were cultured in 250 mL flasks containing 50 mL of half salt strength, basal (free growth regulator) liquid medium (Murashige and Skoog, MS; Caisson Laboratories Inc., Smithfield, UT, United States). All flasks were maintained in a dynamic state using a shaker (JulaboV, Julabo Labortechnik GmBH, D-7633, Seelbach, Germany) for one week. Thereafter, the cultures were transferred to different solid media *viz*; MS, Linsmaier, and Skoog (LS), and Woody Plant (WP) (Caisson Laboratories Inc., Smithfield, UT, United States), either in basal or half-strength, basal composition (M1-M6, Table 2). All media were enriched with 30 g/L sucrose, and 7 g/L agar (WinlabLtd, Leicestershire, UK), then the pH was adjusted to 5.8 using 0.1 N KOH or HCl. All cultures were incubated in complete darkness for one week before being exposed to 3000 lux of light intensity from a cool, white, fluorescent lamp, kept at 26 ± 1 °C with a 16 h photoperiod, and monitored for germination over 28 days of culture. The percentage (%) of germinated seeds (number of germinated seeds/total number of cultured seeds x100) and percentage (%) of seed development (number of survival and developed plantlets/number of cultured seeds x100) were recorded at the end of the cultivation period.

#### 3.1.3 Callus induction

By the end of the 28-day culture period, *P. cattleianum's* seeds were successfully germinated into fully grown plantlets. Consequently, leaves were excised and cultured on free MS solid medium or MS combined with cytokines (C1–C7, Supplementary Table 3**)** such as kinetin (Kin.), and benzyl adenine (BA) (Santa Cruz Biotechnology Inc., Dallas, TX, United States) and auxins as 2,4-dichloro phenoxy acetic acid (2,4-D) (Sigma chemicals, United States). Cultures were incubated under light conditions for 28 days at 25 ± 1 °C. Calli production percentage (Number of explants forming calli/total number of cultured explants × 100), and calli fresh and dry weights (mg/culture), were calculated and recorded.

#### 3.1.4 Growth dynamic and calli mass production

The jar contains one explant with an initial weight ≈of 100 mg/culture) was evaluated for its growth dynamics for five weeks, and the growth index (GI) was calculated as follows: GI= (calli weight on week X - initial calli weight/initial calli weight). Thereafter, the obtained calli were sub-cultured on MS medium supplemented with 3 mg/L BA in combinations with 1 mg/L 2,4-D and 0.2 mg/L Kin (Media C6, Table 3, the best media for callus induction) and the incubation continued for 5 months intervals under the best condition for calli induction aiming to mass-calli production. Both fresh and dry weights (g/jar) were recorded periodically.

#### 3.1.5 Indirect shoot regeneration

The obtained calli were cultured on free MS solid medium or MS media fortified with different concentrations of growth regulators (S1–S5, Table S1**).** All cultures were incubated at 25 ± 1 °C under light conditions provided by white, fluorescent lamps of 1500 lux intensity. The percentage of indirect regeneration, the number of leaves/shoot, shootlets fresh, and dry weights (mg/culture), and average shoot length (cm) were recorded.

#### 3.1.6 Root induction

The obtained shootlets (11 mm^2^) were recultured on free MS solid medium or MS media supplemented with different auxins (R1-R4, Table S2) for root induction. The number of roots/shoot, the average root length (cm), and fresh and dry weights (mg/culture) of formed roots were recorded after 4 weeks.

#### 3.1.7 Acclimatization

The rooted shootlets were washed with distilled water to completely remove the residual solid media. Thereafter, they were disinfected by soaking for 20 min in 1 g/L benlate solution as a fungicidal before being transferred to pots (10 cm) containing sand or peat moss, or a mixture of them (1:1). The pots were covered with clear polyethylene bags for 10 days, then these bags were perforated to allow gas exchange and sprayed with water to maintain high relative humidity. The covers were completely removed after four weeks, and the percentage of acclimatized and surviving plantlets was recorded.

### 3.2 Phytochemical analysis of the phenolic constituents

#### 3.2.1 Extraction of the phenolic constituents

About 0.5 g of *p. cattleianum* original plant, the induced calli, as well as the regenerated shootlets (leaf and stem), were individually extracted with 80 % aqueous methanol (3 x 20 mL). The pooled extracts were separately filtered, dried under reduced pressure at 60 °C, then transferred to pre-weighted vials and kept in the refrigerator for comprehensive phytochemical analysis including the estimation of the total phenolic, flavonoid, and tannin contents in addition to the quantitation of 38 individual phenolic metabolites by High-Performance Liquid Chromatography-Tandem Mass Spectrometry (HPLC-MS/MS).

#### 3.2.2 Determination of total phenolic content (TPC)

It was determined according to the Folin-Ciocalteu colorimetric method [54] and the absorbances were measured at 725 nm. The TPC was calculated from the calibration curve prepared by different concentrations of gallic acid (50–300 μg/mL), and the results were expressed as milligrams of gallic acid equivalent (mg GAE) per gram of dry weight.

#### 3.2.3 Determination of total tannin content (TTC)

It was estimated calorimetrically using the Folin-Ciocalteu method as previously stated [54], except for the calibration curve which was prepared using different concentrations of tannic acid (50–300 μg/mL). The results were expressed as milligrams of tannic acid equivalent (mg TAE) per gram of dry weight.

#### 3.2.4 Determination of total flavonoid content (TFC)

It was established using the aluminum chloride (AlCl_3_) colorimetric protocol stated earlier [54]. The absorbances were measured at 510 nm and the TFC was determined from the calibration curve prepared using different concentrations of catechin (50–300 μg/mL) and expressed as milligrams of catechin equivalent (mg CE) per gram dry weight.

#### 3.2.5 Identification of phenolic constituents using HPLC-MS/MS

##### 3.2.5.1 Reagents and standards

Kaempferol- and quercetin-3-*O*-glucoside analytical standards were supplied by the Phyto Lab GmbH & Co. KG (Vestenbergsgreuth, Germany), while the remaining 36 standard phenolics were purchased from Sigma-Aldrich (Milan, Italy). 99 % formic acid was purchased from Merck (Darmstadt, Germany). Deionized water was filtered using the Milli-Q SP Reagent Water System to give ultrapure water with a resistivity of >18 M cm (Millipore, Bedford, MA, United States). All solutions were filtered on 0.2 μm polyamide filters (Sartorius Stedim, Goettingen, Germany) before usage.

##### 3.2.5.2 Preparation of stock solutions

A stock solution of each standard (1 g/L) was prepared in methanol (HPLC grade, Sigma-Aldrich, Milan, Italy) and stored at 5 °C in stoppered, glass bottles until analysis. Standard's working solutions were freshly prepared at different concentrations by diluting stock solutions with HPLC grade, methanol and filtered using Phenex™ RC 4 mm 0.2 μm syringeless filters (Phenomenex, Castel Maggiore, BO, Italy) just before injection into the HPLC instrument.

##### 3.2.5.3 HPLC-MS/MS analysis of phenolic compounds

The quantification of the phenolic constituents was carried out using a modified version of the previously described method by Mustafa et al. [37]. The dried extracts were dissolved in methanol (5 mg/mL), and sonicated for 2 min at room temperature, then the solutions were filtered using a 0.2 m syringeless filter before being injected into the HPLC–MS/MS system. The HPLC-MS/MS investigations were implemented using an Agilent 1290 Infinity series with triple quadrupole 6420 (Agilent Technology, Santa Clara, California, United States), coupled to an electrospray ionization (ESI) source operated in negative and positive ionization modes. The MS/MS parameters of each standard were optimized using flow injection analysis (FIA). The separation of phenolic compounds was accomplished on a Phenomenex Synergi Polar–RP C18 column (250 mm × 4.6 mm, 4 μm). The mobile system, water (solvent A) and methanol (solvent B) both with 0.1 % formic acid, was prepared and used as follows: 0–1 min in isocratic mode, 20 % B; 1–25 min, gradient mode, 20–85 % B; 25–26 min, isocratic mode, 85 % B; 26–32 min, gradient mode, 85–20 % B. The injection volume was 2 μl, and the flow rate was kept at 0.8 mL/min. The temperature of the column was set to 30 °C, and the drying gas temperature in the ionization source was set to 350 °C. The flow rate of the gas was adjusted to 12 L/min, the capillary voltage was 4000 V and the nebulizer pressure was 55 psi. The peak areas were integrated for quantitation after detection in the dynamic-multiple reaction monitoring (dynamic-MRM) mode. Each analyte's most abundant product ion was employed for quantification, while the other ions were used for qualitative analysis. Each compound's unique time window (Δ retention time) was set at 2 min.

### 3.3 Biological investigations

#### 3.3.1 Cell lines and culture conditions

Human hepatocellular carcinoma (HepG-2), human breast adenocarcinoma (MCF-7), and human leukemia (K-562) cells (American Type Culture Collection, ATCC, Rockville, MD, United States) were used for evaluation of cytotoxic activity, while the murine RAW 264.7 macrophage-like cells (VACSERA (Dokki, Giza, Egypt) was used for investigating the pro- and anti-inflammatory. RPMI-1640 supplemented with 10 % heat-inactivated fetal calf serum and 50 μg/mL gentamycin as well as, Dulbecco's modified eagle medium (DMEM) supplemented with 10 % inactivated fetal bovine serum (Lonza biosciences, Verviers, Belgium), 1 % penicillin-streptomycin (Sigma, St. Louis, Mo., United States) were used for growing cancer cell lines and murine RAW264.7, respectively. All cells were maintained at 37 °C in a humidified atmosphere with 5 % CO_2._

#### 3.3.2 Stock solutions

Tested extracts (original plant, callus, and regenerate plant) were prepared in sterile, biological-grade DMSO (Sigma, St. Louis, Mo., United States) at a final concentration of 1.0 and 0.5 mg/mL for the cytotoxicity, and anti-inflammatory studies, respectively. On the treatment day, each stock solution is serially diluted using the suitable culture medium to prepare final concentrations of 0–1000 and 0–500 μg/mL for the cytotoxicity, and anti-inflammatory studies, respectively. For the antioxidant activity, each extract was prepared in methanol at a final concentration of 1 mg/mL, then serially diluted to prepare final concentrations of 0–1000 μg/mL.

#### 3.3.3 Evaluation of cytotoxicity on cancer cell lines using viability assay

Cells were suspended in RPMI-1640 medium and added to Corning® 96-well culture plates (Corning Inc., New York, United States) at a concentration of 5x10^4^ cells/well. Plates were incubated for 24 h or till the formation of the adherent monolayer, thereafter, media were replaced by fresh ones containing different concentrations of the tested extracts, each in three replicates. Wells containing media alone or 0.5 % sterile, DMSO as vehicle control. After 24 h incubation, the number of viable cells was determined using MTT (3- (4, 5-dimethyl thiazolyl)-2, 5-diphenyltetrazolium bromide, Sigma, St. Louis, Mo., United States) assay as stated previously [55].

#### 3.3.4 Evaluation of the anti-inflammatory effect

##### 3.3.4.1 Evaluation of cytotoxicity on macrophage cells using viability assay

The RAW 264.7 murine macrophage cells were seeded in a 12-well culture plate at a density of 5 x 10^5^ cells/well using Dulbecco's Modified Eagle's Medium (DMEM). Cells were maintained and treated as mentioned above (2.3.3.), while percentage cell viability was determined using an MTT assay following the protocol described previously [56]. The concentration required for 50 % cell growth inhibition (IC_50_) was calculated from the obtained dose-response curves.

##### 3.3.4.2 Determination of pro- or anti-inflammatory activities

RAW 264.7 cells (2 × 10^5^) were treated with the sub-toxic concentration (½ IC_50_) of each tested extract separately as stated earlier by our research team [57]. Three hours later, 100 ng/mL lipopolysaccharide (LPS) was added, and the cells were re-incubated for 24 h at 37 °C. Thereafter, media were removed and centrifuged at 1500×*g* to remove cell debris then the supernatant, was used to evaluate the cytokines as tumor necrosis factor-alpha (TNF-α), interleukin 6 (IL-6), and interleukin 10 (IL-10) using an ELISA assay according to the manufacturer's protocol (R&D Systems® ELISA kit). All incubation steps were performed at room temperature, and the optical densities were measured at 450 nm and compared to the positive control. Cells without any treatment and those treated with LPS served as negative and positive controls, respectively [58].

#### 3.3.5 Evaluation of the antioxidant capacity using 2,2-diphenyl-1-picrylhydrazyl (DPPH) radical scavenging assay

The antioxidant activity of the tested extracts was investigated in terms of DPPH radical scavenging capacity following the procedure stated by Baliyan et al. [59]. Shortly, an aliquot of each tested extract (40 μL) was added to 3 mL of freshly prepared DPPH (0.004 %w/v). Absorbances were recorded immediately at 517 nm on a UV–visible spectrophotometer (Milton Roy, Spectronic 1201). The decrease in absorbance was recorded at zero time, then at 1-min intervals until it stabilized after 16 min. Trolox and DPPH-free solutions were used as positive and negative controls, respectively. The percentage inhibition (PI) of the DPPH radical was calculated according to the formula: PI = [{(A_C_- A_T_)/A_C_} x 100]

Where A_C_ = Absorbance of the control at t = 0 min and A_T_ = absorbance of the tested extract at t = 16 min**.**

### 3.4 Statistical analysis

Data from the micropropagation study were analyzed from three different experiments and expressed as mean ± SE using the Graph Pad Prism Software version 5.0 (La Jolla, CA, United States). Values followed by different superscripts in the same category are significantly different according to Duncan's Multiple Range Test at *p* ≤ 0.05. Biological experiments were implemented in triplicates (n = 3). Reported data represent the calculated mean ± SD. Means were compared using the student t-test, followed by Welch's correction. Values assigned with an asterisk are considered statistically significant relative to control cells at *p* ≤ 0.05.

## 4 Conclusion

Based on the obtained results, it can be concluded that immersion of non-mechanically sacrificed *P. cattleianum* seeds in 15 % commercial bleaching, 15 % HCl, and 10 % H_2_O_2_ minimized the contamination and enhanced the germination percentage. MS medium Fortified with 3 mg BA was found to be the best concentration of cytokinin that promotes callus induction in combination with 1 mg/L 2,4-D + 0.2 mg/L Kin. However, a high percentage of regenerated shootlets was acquired from MS medium combined with 160 mg/L adenine sulfate+0.5 mg/L NAA+0.2 mg/L GA_3._ HPLC-MS/MS revealed the presence of a panel of phenolic compounds in the original and regenerated plants while phenolic profiling of the induced callus derived from polyphenol-rich explants composed of simple phenolic scaffolds, which are then consumed by regenerated plantlets to biosynthesize sophisticated polyphenols. Twenty-eight compounds were identified and quantified in the original plant, while twenty-five were in both regenerated plant and induced callus by HPLC-MS/MS. *P. cattleianum* phenolics’ rich extracts, possessed high anti-inflammatory and antioxidant capacity while displaying minimal cytotoxicity to cancer cells. In all, this study provides a promising approach for the micropropagation of *Psidium cattleianum* Sabine, and the current findings highlight the potential of its *in vitro* propagated plant as a source of bioactive phenolic compounds and support further exploration of its applications in both food and pharmaceutical industries.

## Funding

The grants that supported this work were from the 10.13039/100010002Ministry of Education of Taiwan (DP2-111-21121-01-N-01-03), the National Science and Technology Council of Taiwan (10.13039/501100004663MOST 111-2321-B-255-001, 10.13039/501100004663MOST 110-2320-B-038-034, and 10.13039/501100004663MOST 111-2320-B-038-040-MY3), and 10.13039/501100004700Taipei Medical University (TMU109-AE1-B15).

## Data availability statement

The original contributions presented in the research are included in the manuscript, and further queries can be engaged to the corresponding authors.

## CRediT authorship contribution statement

**Eman M. El-Deeb:** Writing – original draft, Resources, Methodology. **Heba E. Elsayed:** Writing – review & editing, Writing – original draft, Investigation, Data curation. **Hanaa B. Ateya:** Writing – review & editing, Writing – original draft, Software, Methodology, Data curation. **Hussein S. Taha:** Writing – review & editing, Supervision, Methodology, Data curation. **Mohamed R. Elgindi:** Writing – review & editing, Supervision. **Doaa Abouelenein:** Writing – review & editing, Methodology, Investigation, Data curation. **Giovanni Caprioli:** Writing – review & editing, Investigation. **Kuei-Hung Lai:** Writing – review & editing, Methodology, Funding acquisition. **Ahmed M. Mustafa:** Writing – review & editing, Software, Investigation. **Fatma A. Moharram:** Writing – review & editing, Writing – original draft, Supervision, Investigation, Data curation.

## Declaration of competing interest

The authors declare the following financial interests/personal relationships which may be considered as potential competing interests:

Kuei-Hung Lai reports financial support was provided by The 10.13039/100010002Ministry of Education of Taiwan (DP2-111-21121-01-N-01-03). Kuei-Hung Lai reports financial support was provided by the National Science and Technology Council of Taiwan (10.13039/501100004663MOST 111-2321-B-255-001, 10.13039/501100004663MOST 110-2320-B-038-034, and 10.13039/501100004663MOST 111-2320-B-038-040-MY3). Kuei-Hung Lai reports financial support was provided by 10.13039/501100004700Taipei Medical University (TMU109-AE1-B15). If there are other authors, they declare that they have no known competing financial interests or personal relationships that could have appeared to influence the work reported in this paper.
