# Supplementary material for: Phenolic profiling and bioactivity assessment of in vitro propagated Psidium cattleianum Sabine: A promising study
Source: Heliyon. 2024 Apr 9;10(8):e29379. doi: 10.1016/j.heliyon.2024.e29379 (PMC11033136; doi:10.1016/j.heliyon.2024.e29379)
Supplement: Multimedia component 1 [file mmc1.docx]

**Table S1. Effect of various growth regulators supplemented to MS media individually or in combination on indirect shoots regeneration of *P. cattleianum***

| **Media** | **Composition** | **%**  **Regeneration** | **Length of**  **Shoots** | **No. of**  **Shoots** | **No. of Leaves/Shoots** | **Fresh weight**  **(mg/jar)** | **Dry weight**  **(mg/jar)** |
| --- | --- | --- | --- | --- | --- | --- | --- |
| **S1** | MS free; basal | 0^d^ | 0^e^ | 0^c^ | 0^d^ | 0^c^ | 0^c^ |
| **S2** | MS+ 1 mg/L BA+ 160 mg/L AS + 0.5 mg/L NAA+ 0.02 mg/L GA_3_ | 7.33±0.88^c^ | 0.63±0.08^d^ | 1^b^ | 4±0.57^c^ | 470.93±24.74^b^ | 39.40±4.36^b^ |
| **S3** | MS+ 2 mg/L BA+160 mg/L AS+0.5 mg/L NAA+0.02 mg/L GA_3_ | 17.3±1.45^b^ | 1.16±0.14^c^ | 1^b^ | 5±0.57^b,c^ | 574.36±21.29^b^ | 48.60±1.42^b^ |
| **S4** | MS+ 3 mg/L BA+160 mg/L AS+0.5 mg/L NAA+0.02 mg/L GA_3_ | 28.6±3.28^a^ | 2.06±0.12^a^ | 1.66±0.33^a^ | 8.33±0.88^a^ | 760.93±41.02^a^ | 64.73±3.70^a^ |
| **S5** | MS+ 5 mg/L BA+160 mg/L AS+0.5 mg/L NAA+0.02 mg/L GA_3_ | 24±2.08^a^ | 1.53±0.12^b^ | 1^b^ | 6.66±0.66^a,b^ | 851.73±62.90^a^ | 73.13±4.38^a^ |

BA: Benzyl adenine; AS: Adenine sulphate; NAA: Naphthalene acetic acid, GA3: Gibberellic acid; Data are represented as means ±SE. Values followed by different superscripts in the same column differ significantly according to Duncan’s Multiple Rang Test (DMRT) at p≤0.05. (Cultures were incubated for 4 weeks under light conditions (16/8 h) and 1200 Lux from florescent white lamps for 4 subcultures)

**Table S2. Effect of various auxins supplemented to MS media** on percentage of root formation, root length (cm), roots number, **fresh and dry weights (mg/ jar) of *P. cattleianum* Sabine**

| **Media** | **Composition** | **%Root formation** | **Length of root (cm)** | **No. of root/shoot** | **Fresh weight(mg/jar)** | **Dry weight(mg/jar)** |
| --- | --- | --- | --- | --- | --- | --- |
| **R1** | MS free; basal | 37±2.64^c^ | 3.46±0.33^c^ | 0.66±0.33^a^ | 156.53±2.53^c^ | 15.33±0.26^c^ |
| **R2** | MS+1mg/L IBA | 76±4.35^a^ | 6±0.17^a^ | 1.66±0.33^a^ | 557.83± 50.34^a^ | 46.80±5.11^a^ |
| **R3** | MS+1mg/L IAA | 59.33±1.85^b^ | 4.70±0.23^b^ | 1.33±0.33^a^ | 461.56±33.08^a^ | 36.16±3.69^b^ |
| **R4** | MS+1mg/L NAA | 42.33±2.02^c^ | 4.46±0.14^b^ | 1.00^a^ | 318.30±34.79^b^ | 22.33±1.54^c^ |

IBA: [Indole-3-butyric acid, IAA: Indole-acetic acid, NAA: Naphthalene acetic acid. Data are represented as means ±SE. Values followed by different superscripts in the same column are differ significantly according to Duncan’s Multiple Rang Test (DMRT)at p≤0.05.](https://www.plantcelltechnology.com/indole-3-butyric-acid-iba-solution/)[(Cultures were incubated for 4 weeks under light conditions (16/8 h) for 4 subcultures)](https://www.plantcelltechnology.com/indole-3-butyric-acid-iba-solution/)

**Table S3. Effect of different soil composition on plantlet acclimatization and survival percentage**

| **Soil type** |  | **Acclimatization%** |
| --- | --- | --- |
| Peatmoss |  | 18±2.1^b^ |
| Peatmoss+sand (1:1) |  | 33.6±2.4^a^ |
| Sand |  | 5.3±1.4^c^ |

Data are represented as means ±SE. Values followed by different superscripts in the same column are differ significantly according to Duncan’s Multiple Rang Test (DMRT) at p≤0.05

**Table S4. HPLC–MS/MS** **acquisition parameters (dynamic-MRM mode) used for the analysis of the 38 marker compounds.**

| **Compounds** | **Precursor**  **ion, *m/z*** | **Product**  **ion, *m/z*** | **Fragmentor, V** | **Collision energy, V** | **Polarity** | **R_t_** |
| --- | --- | --- | --- | --- | --- | --- |
| Gallic acid | 169 | 125.2^*^ | 97 | 12 | Negative | 6.96 |
| Neochlorogenic acid | 353 | 191.2^*^, 179 | 82 | 12, 12 | Negative | 9.52 |
| Delphinidin-3-galactoside | 465.01 | 303^*^ | 121 | 20 | Positive | 11.36 |
| (+)-Catechin | 289 | 245.2^*^,109.2 | 131 | 8, 20 | Negative | 11.44 |
| Procyanidin B2 | 576.99 | 576.99^*^, 321.2 | 160 | 0, 32 | Negative | 12.41 |
| Chlorogenic acid | 353 | 191.2^*^, 127.5 | 82 | 12, 20 | Negative | 12.42 |
| *p*-Hydroxybenzoic acid | 137 | 93.2^*^ | 92 | 16 | Negative | 12.86 |
| (-)-Epicatechin | 289 | 245.1^*^, 109.1 | 126 | 8, 20 | Negative | 13.03 |
| Cyanidin-3-glucoside | 449 | 287.3^*^, 255.6 | 121 | 20, 20 | Positive | 13.14 |
| Petunidin-3-glucoside | 479.01 | 317^*^, 302 | 121 | 20, 44 | Positive | 13.26 |
| 3-Hydroxybenzoic acid | 137 | 93.2^*^ | 88 | 8 | Negative | 13.59 |
| Caffeic acid | 179 | 135.2^*^, 134.1 | 92 | 12, 24 | Negative | 13.65 |
| Vanillic acid | 167 | 152.4^*^, 108.1 | 88 | 12, 20 | Negative | 14.32 |
| Resveratrol | 227 | 185^*^, 143.2 | 136 | 12, 20 | Negative | 14.40 |
| Pelargonidin-3-glucoside | 433.01 | 271^*^, 121 | 116 | 24, 50 | Positive | 14.52 |
| Pelagonidin-3-rutinoside | 579.01 | 271^*^ | 145 | 32 | Positive | 14.56 |
| Malvidin-3-galactoside | 493.01 | 331^*^, 315.1 | 121 | 20, 50 | Positive | 14.64 |
| Syringic acid | 196.9 | 182.2^*^, 121.2 | 93 | 8, 12 | Negative | 15.28 |
| Procyanidin A2 | 575 | 575^*^, 285 | 170 | 0, 20 | Negative | 16.18 |
| *p*-Coumaric acid | 163 | 119.2^*^, 93.2 | 83 | 12, 36 | Negative | 16.70 |
| Ferulic acid | 193 | 134.2^*^, 131.6 | 83 | 12, 8 | Negative | 17.10 |
| 3,5-Dicaffeoylquinic acid | 514.9 | 353.1^*^, 191 | 117 | 8, 28 | Negative | 17.61 |
| Rutin | 609 | 300.2^*^, 271.2 | 170 | 32, 50 | Negative | 17.73 |
| Hyperoside | 465.01 | 303^*^, 61.1 | 97 | 8, 50 | Positive | 18.33 |
| Isoquercitrin | 463 | 271.2^*^, 300.2 | 155 | 44, 24 | Negative | 18.36 |
| Delphinidin-3,5-diglucoside | 462.9 | 300.1^*^ | 165 | 24 | Negative | 18.38 |
| Phloridzin | 435.39 | 273^*^, 167 | 155 | 8, 28 | Negative | 18.83 |
| Quercitrin | 446.99 | 300.2^*^, 301.2 | 160 | 24, 16 | Negative | 19.61 |
| Myricetin | 316.99 | 179.1^*^, 182 | 150 | 16, 24 | Negative | 19.61 |
| Naringin | 578.99 | 271.3^*^, 151.3 | 170 | 32, 44 | Negative | 19.62 |
| Kaempferol-3-glucoside | 447 | 284.2^*^, 255.2 | 170 | 24, 40 | Negative | 19.77 |
| Hesperidin | 611.01 | 303^*^, 334.8 | 112 | 20, 12 | Positive | 20.19 |
| Ellagic acid | 301 | 301^*^, 229 | 170 | 0, 24 | Negative | 21.41 |
| *Trans*-Cinnamic acid | 149 | 131.2^*^, 77.2 | 74 | 4, 36 | Positive | 21.44 |
| Quercetin | 300.99 | 151.2^*^, 179.2 | 145 | 16, 12 | Negative | 21.87 |
| Phloretin | 272.99 | 167^*^, 123 | 116 | 8, 20 | Negative | 22.30 |
| Kaempferol | 287.01 | 153^*^, 69.1 | 60 | 36, 50 | Positive | 23.84 |
| Isorhamnetin | 314.99 | 300.2^*^, 196.1 | 145 | 16, 4 | Negative | 24.57 |

R_t_ Retention time in min; ^*^These product ions were used for quantification.

Separation condition: The mobile system, water (solvent A) and methanol (solvent B), both with 0.1% formic acid, was prepared and used as follows: 0–1 min in isocratic mode, 20% B; 1–25 min, gradient mode, 20–85% B; 25–26 min, isocratic mode, 85% B; 26–32 min, gradient mode, 85–20% B. The injection volume was 2 μl, and the flow rate was kept at 0.8 mL/min. The temperature of the column was set to 30 °C, and the drying gas temperature in the ionization source was set to 350 °C. The flow rate of the gas was adjusted to 12 L/min, the capillary voltage was 4000 V and the nebulizer pressure was 55 psi. The peak areas were integrated for quantitation after detection in the dynamic-multiple reaction monitoring (dynamic-MRM) mode. Each analyte's most abundant product ion was employed for quantification, while the other ions were used for qualitative analysis. Each compound's unique time window (Δ retention time) was set at 2 min.

| **(A) (B) (C)**  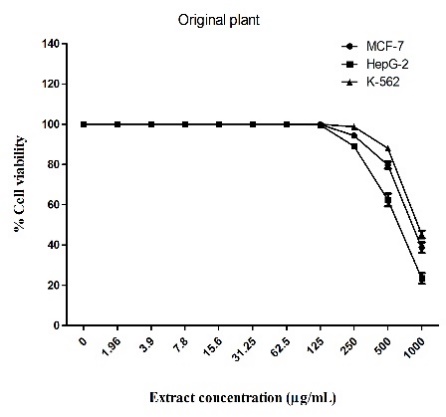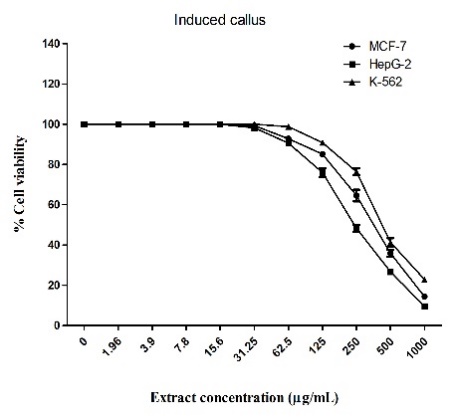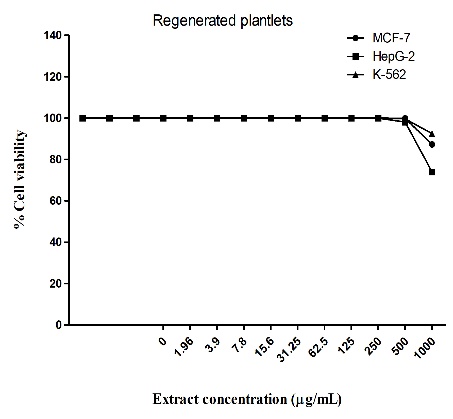 |
| --- |

**Figure S1: Dose response effect of *P. cattleianum* Sabine extracts obtained from (A) original plant (B) callus (C) regenerated plantlet on the viability of HepG-2, MCF-7 and K-562**
